# Association Between Gastrointestinal Symptoms and Disease Severity in Multiple Sclerosis Patients

**DOI:** 10.7759/cureus.83386

**Published:** 2025-05-03

**Authors:** Fajer Saleem, Sajjal Shadia, Vaisnavy Govindasamy, Rehab Fatima, Ramla M Khan, Keziah Nithiya Suresh, Steve Paulson John, Sana Khalid, Tamer Azzam Fuad Mubarak, Mohamed Khalafalla Adlan Abdelsadig, Sher Bano

**Affiliations:** 1 General Medicine, Ayub Medical College Abbottabad, Abbottabad, PAK; 2 General Medicine, Shifa College of Medicine, Islamabad, PAK; 3 Internal Medicine, South Tees Hospital NHS, Middlesbrough, GBR; 4 General Medicine, Shifa Tameer-E-Millat University, Shifa College of Medicine, Islamabad, PAK; 5 Internal Medicine, Thumbay University Hospital, Medicity, ARE; 6 General Medicine, Thumbay University Hospital, Ajman, ARE; 7 Pathology, Rawalpindi Medical University, Lahore, PAK; 8 Internal Medicine, Rashid Hospital, Dubai, ARE; 9 Internal Medicine, University Hospital Waterford, Waterford, IRL; 10 Clinical Psychology, NeuroWave Research Center, Islamabad, PAK

**Keywords:** chronic illness, disease severity, gastrointestinal symptoms, multiple sclerosis (ms), neurodegenerative disease

## Abstract

Introduction

Multiple sclerosis (MS) is a chronic autoimmune neurodegenerative disease that typically leads to neurological signs along with physical and cognitive issues while affecting the gastrointestinal (GI) system. GI dysfunctions like constipation and indigestion frequently affect MS patients, although their relationship with MS disease progression is not well documented.

Methods

The research team conducted a cross-sectional quantitative study that enrolled 300 adult MS patients between 18 and 65 years of age through purposive sampling. Data collection occurred from January through March 2025 using standardized tools for self-report assessments, clinical evaluations (Gastrointestinal Symptom Rating Scale (GSRS) and Expanded Disability Status Scale (EDSS)), and demographic information. Statistics in this study used Pearson correlation and linear regression methods to establish the relationship between GI symptoms and MS severity determinants. The analysis employed one-way ANOVA for group means assessment according to age categories alongside treatment facilities, followed by chi-square (χ²) for examining age-oriented treatment center relations.

Results

The research data revealed significant positive links between MS disease severity levels and gastrointestinal symptoms (GI) severity (r = 0.132, p <0.05). In contrast, GI symptoms emerged as an essential predictor of disease severity (β = 0.148, p <0.01). The age groups did not affect GI symptoms significantly (F (2,297) = 0.326, p > 0.05). Government centers had higher levels of disability and GI symptoms (F (3,296) = 14.92, p <0.01). The type of treatment center showed no correlation with the age distributions of patients (χ² = 3.525, p = 0.474). The study participants most commonly fell into the age range of 22-24 years old (198 participants (66%)), while 73% (218 participants) were male. Government-controlled treatment centers indicated more gastrointestinal symptoms as well as disability levels when analyzed against both private facilities and semi-government centers.

Conclusion

The research confirms that gastrointestinal symptoms are both relevant and very weakly associated with disease progression in MS. Systematically observing gastrointestinal symptoms in MS management will generate better patient treatment outcomes. Long-term investigations about treatment strategies must be conducted to measure cause-and-effect relationships while perfecting symptom management strategies for MS patients.

## Introduction

The most common cause of disability in young adults is multiple sclerosis (MS), and there is an increasing incidence around the world due to various gene-environmental interactions [1]. MS is an autoimmune neurodegenerative disorder referring to both physical and cognitive impairments resulting from inflammatory demyelination and neurodegeneration, which are mainly caused by microglial activation, oxidative stress, and mitochondrial dysfunction [2,3]. Recently acquired knowledge shed light on the prominent role of B cells among other entities and focused upon the greater efficacy that high-efficacy therapies can achieve, especially when begun early [2].

The diagnosis of MS must be at the earliest stage possible for specific treatment, which focuses on the management of relapsing forms while taking care of a progressive disease [4]. MS stands as one of the autoimmune neurodegenerative diseases in young adults, which causes physical and cognitive impairment. It is treated by medications that decrease the relapse rate by 29% to 68% [5]. Current research on MS indicates that immune cells at different stages of disease have shared functions in disease development, highlighting the potential for therapeutic manipulation of immune cell plasticity to provide new therapeutic avenues [6].

The financial cost of MS grows with the severity of the disease, with mounting costs for healthcare and reduced quality of life as a function of the healthcare system structure [7]. MS is a significant cause of neurological disability among young and middle-aged adults, with demyelinating plaques in the central nervous system, characterized by unpredictable symptoms with variable severity [8]. Progressive MS is linked with increased lesion load and more significant numbers of mixed active/inactive lesions, which may be used as prognostic indicators for disease progression [9].

New evidence also points toward gastrointestinal involvement in MS, with increased visits to the doctor and the use of drugs related to the GI tract in the last five years before diagnosis, indicating a prodromal phase [10,11]. Most cases of MS with gastrointestinal complications produce symptoms including constipation and difficulties swallowing, along with food control issues and stomach discomfort [12]. Some diseases, like Guillain-Barré syndrome and MS, exhibit similar neuroinflammation mechanisms that may culminate in gastrointestinal problems usually appearing before or simultaneously with neurologic manifestations [13]. In animal studies, after tests of the experimental autoimmune encephalomyelitis (EAE), to show dysmotility of the gastrointestinal tract, and some autoantibodies targeting enteric nervous system structures support some correlation between autoimmune reaction and GI symptomatology [14].

Gastrointestinal dysfunction in the form of constipation and delayed gastric emptying is common in individuals affected by MS. They often appear concomitantly with bladder symptoms [15]. MS may be a cause of autonomic nervous system dysfunction, leading to gastrointestinal, urological, and cardiovascular disturbances that require early detection and definitive treatment modalities [16]. Such multiple system involvement calls for early diagnosis for management and holistic measures. Acquiring insight into gastrointestinal symptoms connections to MS severity helps clinicians provide better patient care and aids researchers in understanding disease progression mechanisms. The investigation of these relationships provides healthcare providers with better knowledge to create comprehensive treatment approaches for MS patients while also enhancing their symptom management capabilities.

Rationale

The central nervous system disease MS shows various clinical symptoms, which include cognitive difficulties alongside disabilities of movement. Medical assessments now show that more patients with MS experience gastrointestinal complications, which include bowel movement problems and abdominal discomfort. Many patients experience these symptoms, but medical professionals commonly underestimate both their reports and their descriptions within MS cases.

The gastrointestinal system of MS patients experiences major problems that decrease both their ability to function during the day and their quality of life. Little study has been conducted to link GI symptom intensities with the progression trajectory of MS, even though these symptoms commonly appear. This research evaluates how closely gastrointestinal symptom intensity relates to the total MS disease severity in patients with MS.

The investigation evaluates the occurrence of gastrointestinal symptoms in MS patients by analyzing their relationship with Expanded Disability Status Scale (EDSS) scores. Further comprehension of this connection holds crucial importance because it allows healthcare providers to create comprehensive care plans that cover neurological symptoms and other aspects of MS. The new data provides doctors with an extended patient perspective about MS effects, which thus helps them create individualized care plans that deliver better outcomes. The outcomes from this research can guide future investigations focused on therapeutic approaches for decreasing MS patients' gastrointestinal problems through diet modifications or medicine adjustments, and gut motility or gut microbiota treatments.

The research results have the potential to enhance medical recognition of gastrointestinal symptoms as fundamental elements of MS treatment while improving patient experiences and outcomes. Understanding both gastrointestinal symptoms and their relationship to disease severity among MS patients will expand patient care strategies throughout the MS community because these symptoms usually receive inadequate diagnoses and reports.

Objectives

This study’s main goal was to analyze how GI symptom intensity relates to MS disease severity. The study investigated multiple variables that affect the association between GI symptom severity and MS severity. Study participants received EDSS testing for MS severity analysis while researchers evaluated both the commonness and nature of GI symptoms in MS patients.

## Materials and methods

Study design and setting

A cross-sectional quantitative research study analyzed the association between GI symptoms and MS disease severity in adult patients. The main aim of the research was to evaluate how frequently MS patients experience gastrointestinal symptoms and whether symptom intensity is related to disease severity in MS patients.

The research selected (N=300) MS patients between 18 and 65. The study recruited patients from a hospital environment to ensure adequate medical oversight and access to their current diagnostic and therapeutic records. This setting enabled proper clinical screening procedures in combination with standardized data collection protocols.

Participants needed to meet two conditions: First, they needed a confirmed MS diagnosis, and second, they had to be capable of providing voluntary consent. Participants with significant cognitive impairment, other neurological diseases besides MS, or gastrointestinal disorders unrelated to MS (such as irritable bowel syndrome or inflammatory bowel disease) were not included in the study. Data was collected from January 2025 to March 2025.

Sample size and technique

An estimated (N=300) number of participants was needed to satisfy WHO sample size calculations based on a 95% confidence level, a population proportion of 0.90, and an absolute precision of 0.05. Our estimated sample size was 300. A purposive sampling technique was employed to recruit participants who met the inclusion criteria. This non-probability sampling method was appropriate for the study as it allowed the selection of individuals with confirmed MS diagnoses and relevant clinical profiles.

Data collection tools and procedure

The data collection process included using several standardized instruments to evaluate GI symptoms and disease severity in MS patients. Patients used the validated patient self-report tool, the Gastrointestinal Symptom Rating Scale (GSRS) (Appendix A), to measure their gastrointestinal symptoms according to their perceived intensity. It was developed by Jan Svedlund, Ingemar Sjödin, and Gerhard Dotevall in 1988. The GSRS measures frequent GI symptoms like constipation, bloating, pain in the abdomen, diarrhea, and indigestion, each of which is scored on a Likert scale from 1 (no symptoms) to 7 (severe symptoms). The internal consistency reliability for the GSRS scales ranged from 0.61 to 0.83 (Table 6) [17]. The EDSS (Appendix B) measures neurological examination comprehensively. It is a scale ranging from 0 (no disability) to 10 (as per MS death), reflecting various MS features, such as motor function, coordination, sensory function, etc. The greater the number on a scale of 1 to 10, the greater the degree of disability. It was developed by John F. Kurtzke in 1983 (Table 7) [18]. Patient interviews and medical record reviews were data collection methods for obtaining demographic information and clinical data consisting of patient age, gender, illness duration, disease classification (Relapsing-remitting MS, secondary progressive MS, primary progressive MS), and prescribed treatments.

All participants were requested to consent before data collection, ensuring voluntary participation and confidentiality. The data were gathered from various hospitals and clinics where the participants filled out the questionnaires, and the clinician evaluated the EDSS. It allowed for integrated data collection, subjective reports (GI symptoms), and objective clinical assessments (EDSS) for an in-depth analysis of the association of GI symptoms and MS disease severity. The Institutional Review Board of NeuroWave Research Center, Islamabad, Pakistan, approved it with IRB-2025-0098 before starting the research. IBM SPSS Statistics version 26 software (IBM Corp., Armonk, NY, USA) was utilized for analysis. This research used descriptive statistics to provide statistical information about participant demographics and clinical indicators, including participant age, gender, MS classification, and whether they received treatment. Pearson correlation analysis was used to study associations between gastrointestinal symptoms and disease severity, and linear regression analysis was used to establish whether gastrointestinal symptoms contribute significantly to MS severity. One-way ANOVA analyzed the means across groups divided by age and treatment facility regarding their scores on the Gastrointestinal Symptom Rating Scale and the EDSS. A chi-square (χ²) test analyzed the connection between age categories and the treatment centers they chose, between private, government, and semi-government. The significance threshold was established as a p-value of less than 0.05.

## Results

Table 1 shows the demographics of 300 participants with MS. The participant sample was mainly composed of individuals within the age ranges of 22 to 24 years (66%) and 25 to 27 years old (25%), followed by those of 19 to 21 years old (9%). The study population consisted mainly of men, at 218 or 73%, while women accounted for 82 or 27% of participants. The divorce and separation experience among participants reached a combined proportion of 58% (93+82). Divorce and separation rates may indicate how illness affects relationships. The education profile included 82 participants (27%) who graduated from secondary school and 73 (24%) who hold a bachelor’s degree, along with 25 individuals (8%) who pursued a master’s degree. The participants' employment status indicated that 70 students made up 23% of the population, while 64 were unemployed and accounted for 21%, and 59 who were retired made up 20%. Among the respondents, the largest income group reported earning Pakistani rupee (PKR) 25,000-50,000 each month (129 (43%)), but 103 participants (34%) received less than PKR 25,000. Out of 300 individuals with MS, 230 (77%) received their diagnosis between one and two years ago. The study sample included 131 (44%) participants with primary progressive MS, 56 (19%) individuals with secondary progressive MS, and 21 (7%) people with relapsing-remitting MS. Subsequently, 92 (31%) patients remained uninformed about their MS type.

**Table 1:** Demographic characteristics of participants (N=300) f: frequency

Variable	f	%
Age	-	-
19-21 Years	27	9
22-24 Years	198	66
25-27 Years	75	25
Gender	-	-
Male	218	73
Female	82	27
Marital status	-	-
Single	30	10
Married	72	24
Separated	93	31
Divorced	82	27
Widowed	23	8
Educational level	-	-
Primary school (Grades 1-5)	31	10
Secondary school (Grade 6-10)	82	27
Higher Secondary (FA/FSc or equivalent)	73	24
Bachelor’s	73	24
Master’s	25	8
No formal education	16	5
Employment Status	-	-
Student	70	23
Employed (part-time)	52	17
Employed (full-time)	35	12
Unemployed	64	21
Homemaker	20	7
Retired	59	20
Monthly income	-	-
Less than 25,000 Pakistani rupees	103	34
25,000-50,000 Pakistani rupees	129	43
50,000-100,000 Pakistani rupees	45	15
More than 100,000 Pakistani rupees	23	8
Duration since multiple sclerosis diagnosis	-	-
1-2 years	230	77
3-4 years	57	19
5-6 years	13	4
Multiple sclerosis type	-	-
Primary progressive multiple sclerosis	131	44
Secondary progressive multiple sclerosis	56	19
Relapsing-remitting multiple sclerosis	21	7
Don’t know	92	31
Current medication	-	-
Dimethyl fumarate	78	26
Fingolimod	77	26
Steroid treatment for relapses	22	7
Physiotherapy or rehabilitation	50	17
Glatiramer acetate	40	13
Other	17	6
Not currently receiving treatment	16	5
Treatment center	-	-
Private	191	64
Government	53	18
Semi-government	56	19

Table 2 displays the intercorrelation between gastrointestinal symptoms and disease severity in MS patients. The link between scores from the GI Symptom Rating Scale and the EDSS Scale showed a minimum positive correlation (r = 0.132, p <.05) that achieved statistical significance. Evidence shows that more severe gastrointestinal symptoms lead to less disease severity, but the link remains weak.

**Table 2:** Intercorrelations between study variables *p<0.05 considered significant.

Variable	1	2
The Gastrointestinal Symptom Rating Scale	-	0.132^*^
The Expanded Disability Status Scale	0.132^*^	-

Table 3 demonstrates the results of linear regression analysis that evaluates gastrointestinal symptom impacts on disease severity for patients with MS. Health evaluations on gastrointestinal symptoms showed positive alignment with disease severity (B = 0.055, β = 0.148, p <.01) through an interval range from 0.01 to 0.09. People who score higher on gastrointestinal symptom ratings show increased EDSS scores, which indicates higher disability severity. Gastrointestinal symptoms exhibit a less significant relationship, which suggests they contribute to clinical disability evaluation in MS patients.

**Table 3:** Linear regression: Gastrointestinal symptoms and disease severity in multiple sclerosis β: Standardized Coefficient; CI: Confidence Interval, SE: Std. Error; β: Unstandardized Coefficient. *p <0.05. **p <.001. **p <0.001.

Predictor	B	95% Cl		SE	β
		LL	UL		
Constant	15.76	13.1	18.4	1.33	-
The Gastrointestinal Symptom Rating Scale	0.055^**^	0.01	0.09	0.02	0.148^**^

Table 4 analyzes gastrointestinal symptoms in conjunction with disability status, presenting the findings from ANOVA across various treatment centers and age groups. Statistical analysis revealed that age groups have no significant impact on gastrointestinal symptoms, as indicated by a value of F (2,297) = 0.326 (p > 0.05). The 22-24 age group showed the highest reported disability compared to other age groups, with F (2,297) = 10.46, p <.01, statistical significance. The gastrointestinal symptom experience at government treatment centers stood out from other locations since patients at these centers reported the highest symptoms (F (3,296) = 3.24, p < 0.05). The disability status evaluation between treatment facilities revealed significant results (F (3,296) = 14.92, p < 0.01), and government centers displayed increased disability levels. Data indicates that treatment facilities, together with patients' ages, affect disability outcomes rather than their gastrointestinal symptoms in individuals dealing with MS.

**Table 4:** Comparison of Variables (Age and Treatment Center) M:mean; S.D.: standard deviation; F: F-ratio; η2=effect size

Variable	19-21 years	22-24 years	25-27 years	F (2,297)	η2
	M±S.D	M±S.D	M±S.D		
The Gastrointestinal Symptom Rating Scale	62.6±9.12	62.5±8.59	61.6±9.23	0.326	-
The Expanded Disability Status Scale	16.6±2.63	19.6±3.09	19.0±3.42	10.46^**^	0.07
Variable	Private	Government	Semi-government	F (3,296)	η2
	M±S.D.	M±S.D.	M±S.D.		
The Gastrointestinal Symptom Rating Scale	61.3±8.65	64.4±9.24	63.4±8.45	3.24^*^	0.032
The Expanded Disability Status Scale	18.4±2.99	20.5±3.03	20.4±3.49	14.92^**^	0.131

Table 5 displays age population data of treatment participants through different facility types (private, government, and semi-government which undergoes chi-square analysis to validate associations. Participants who fell into the age group of 22-24 years showed the highest participation rates in every facility. The chi-square value from the 19-21-year age category resulted in χ² = 3.525, which produced a non-significant p-value at p = 0.474, revealing no statistical relationship between patients’ age and their chosen treatment setting. There is no statistically substantial connection between treatment center types and the age group composition of patients.

**Table 5:** Descriptive statistics of demographic variables f: frequency; %: percentage; p: level of significance p-values calculated using the chi-square test; the significance level is set at p < 0.05.

Variables	f	Private	Government	Semi-government	p	χ^2^
Age	-	-	-	-	-	-
19-21 years	27	20	4	3	0.474	3.525
22-24 years	198	125	32	41	-	-
25-27 years	75	46	17	12	-	-

## Discussion

This research investigated GI symptom patterns relative to MS disease severity in MS patients because previous research did not adequately study this subject. Our study participant population revealed a different gender balance than global MS patterns because male patients outnumbered female patients. The gender-based pattern of MS development contrasts with typical patterns because women typically demonstrate higher rates of vulnerability to MS primarily due to genetic and hormonal factors [19,20].

The research findings match previous studies showing that chronic illness significantly affects personal relationships. A significant portion of the studied participants were either separated or divorced due to the impact of living with MS on their relationships [21].

Our research indicated a weak yet statistically significant positive relationship between gastrointestinal syndrome severity and MS disease intensity. Prior research has confirmed that gastrointestinal problems frequently manifest in MS patients [12]. The modest link between gastrointestinal symptoms and MS severity stands out from new research, even though more extensive evaluation is required to study this potential relationship.

In line with these findings, linear regression analysis could further confirm that an increase in GI symptom scores could significantly predict that the disease would become more severe. This further strengthens the premise that GI disturbances should not be considered isolated complaints, but possible contributors or one of the indicators of disease progression. Earlier work has demonstrated evidence that lesions in the autonomic centers can lead to very significant gastrointestinal disruption, which, in turn, would cause an increase in the burden of neurological symptoms in MS patients [22].

The research discovered that gastrointestinal symptom intensities remained statistically similar across different age groups, though young participants between 22 and 24 showed more disability manifestations. Previous studies support these findings by showing both GI symptom severity and age, along with depression and anxiety, as essential factors that influence disability outcomes [23,24]. This study demonstrated that treatment centers operated by the government reported more GI symptoms along with superior disability levels than facilities in other settings, thus indicating that disability outcomes depend on patient age and treatment environment. Research findings from previous studies validate that the environment in which treatment takes place remains crucial for determining health results [25].

The findings revealed that patients’ age failed to establish a significant correlation with their treatment facility selection, leading researchers to conclude that patient age does not impact their chosen facility type. Past research has demonstrated that the age of patients does not substantially impact their healthcare utilization behaviors [26].

The research presents multiple limitations that affect the understanding of the recorded results. The results from the cross-sectional study restrict researchers from identifying direct cause-and-effect relationships between gastrointestinal symptoms and MS disease severity. The use of self-reported gastrointestinal symptoms represents potential risks of distorted reporting from participants because their perceptions could differ from true clinical findings. The research sample selection method through convenience sampling fails to represent the general population of patients with MS since it primarily includes participants from specific areas. Many study participants did not know their MS subtype because of which symptom profiles could not be adequately analyzed based on MS classification categories. The study failed to consider psychological elements such as depression and nervousness because these factors affect both digestive system responses and MS symptom perception.

Researchers need to study gastrointestinal symptoms through repeated surveys over time to recognize their role as possible early signs of worsening disability among people with MS. Future studies should combine direct clinical GI tests and patient-reported outcomes to boost research strength.

In the future, specific MS subtypes need to be studied regarding their distinct symptom patterns as well as their separate treatment requirements associated with gastrointestinal symptoms. Additionally, studying how different therapies for MS affect gastrointestinal function will provide knowledge about side effects and advantages that exist in modern treatment approaches. The evaluation of mental health involvement in GI symptom-MS severity relationships requires psychological assessments to be integrated into further research. The general ability of results to apply to different groups will become stronger when studies investigate broader variations of populations through multi-center studies regarding holistic MS care for patients.

## Conclusions

The research shows that GI symptoms demonstrate an essential association with MS disease advancement among people with MS. Research data indicates that patients show higher gastrointestinal problems when their disability level increases, thus demonstrating a connection between autonomic dysfunction and disease advancement. The weak correlation between disease severity and GI symptoms was statistically valid enough to show their clinical value in MS care. Professional assessments of gastrointestinal health during regular care practice help develop more complete patient-focused approaches to healthcare. Additional research studying both the causal factors and temporal link between autonomic dysfunction and MS would help establish better treatment strategies for improved patient outcomes.

## Appendices

**Table 6:** Gastrointestinal Symptom Rating Scale (GSRS) Developed by Svedlund et al. (1988) [17]

Items	Responses
1. Have you been bothered by pain or discomfort in your upper abdomen or the pit of your stomach?	-
2. Have you been bothered by heartburn?	-
3. Have you been bothered by acid reflux?	-
4. Have you been bothered by hunger pains in the stomach?	-
5. Have you been bothered by nausea?	-
6. Have you been bothered by rumbling in your stomach?	-
7. Has your stomach felt bloated?	-
8. Have you been bothered by burping?	-
9. Have you been bothered by passing gas or flatus?	-
10. Have you been bothered by constipation?	-
11. Have you been bothered by diarrhea?	-
12. Have you been bothered by loose stools?	-
13. Have you been bothered by hard stools?	-
14. Have you been bothered by an urgent need to have a bowel movement?	-
15. When going to the toilet, have you had the sensation of not completely emptying the bowels?	-

**Table 7:** Expanded Disability Status Scale (EDSS) FS: Functional Systems; MS: multiple sclerosis Developed by Kurtzke (1983) [18]

Items	Response
No disability	-
No disability, minimal signs in one FS	-
No disability, minimal signs in more than one FS	-
Minimal disability in one FS	-
Mild disability in one FS or minimal disability in two FS	-
Moderate disability in one FS, or mild disability in three or four FS. No impairment to walking	-
Moderate disability in one FS and more than minimal disability in several others. No impairment to walking	-
Significant disability but self-sufficient and up and about some 12 hours a day. Able to walk without aid or rest for 500 m	-
Significant disability but up and about much of the day, able to work a full day, may otherwise have some limitation of full activity or require minimal assistance. Able to walk without aid or rest for 300 m	-
Disability severe enough to impair full daily activities and ability to work a full day without special provisions. Able to walk without aid or rest for 200 m	-
Disability severe enough to preclude full daily activities. Able to walk without aid or rest for 100 m	-
Requires a walking aid - cane, crutch, etc. - to walk about 100 m with or without resting	-
Requires two walking aids - pair of canes, crutches, etc. - to walk about 20 m without resting	-
Unable to walk beyond approximately 5m even with aid. Essentially restricted to wheelchair; though wheels self in standard wheelchair and transfers alone. Up and about in wheelchair some 12 hours a day	-
Unable to take more than a few steps. Restricted to wheelchair and may need aid in transferring. Can wheel self but cannot carry on in standard wheelchair for a full day and may require a motorized wheelchair	-
Essentially restricted to bed or chair or pushed in wheelchair. May be out of bed itself much of the day. Retains many self-care functions. Generally has effective use of arms	-
Essentially restricted to bed much of day. Has some effective use of arms, retains some self-care functions	-
Confined to bed. Can still communicate and eat	-
Confined to bed and totally dependent. Unable to communicate effectively or eat/swallow	-
Death due to MS	-
